# Health Equity Talk: Understandings of Health Equity among Health Leaders

**DOI:** 10.3934/publichealth.2017.5.490

**Published:** 2017-11-15

**Authors:** Bernadette M. Pauly, Sana Z. Shahram, Phuc T. H. Dang, Lenora Marcellus, Marjorie MacDonald

**Affiliations:** 1 Centre for Addictions Research of BC, University of Victoria, PO Box 1700 STN CSC, Victoria, BC, Canada; 2 School of Nursing, University of Victoria, PO Box 1700 STN CSC, Victoria, BC, Canada

**Keywords:** health equity, health equity lens, population lens, social determinants of health, complexity, intersectionality, public health systems and services, constant comparative analysis

## Abstract

**Introduction:**

Reducing health inequities is a stated goal of health systems worldwide. There is widespread commitment to health equity among public health leaders and calls for reorientation of health systems towards health equity. As part of the Equity Lens in Public Health (ELPH) program of research, public health decision makers and researchers in British Columbia collaborated to study the application of a health equity lens in a time of health system renewal. We drew on intersectionality, complexity and critical social justice theories to understand how participants construct health equity and apply a health equity lens as part of public health renewal.

**Methods:**

15 focus groups and 16 individual semi-structured qualitative interviews were conducted with 55 health system leaders. Data were analyzed using constant comparative analysis to explore how health equity was constructed in relation to understandings and actions.

**Results:**

Four main themes were identified in terms of how health care leaders construct health equity and actions to reduce health inequities: (1) population health, (2) determinants of health, and (3) accessibility and (4) challenges of health equity talk. The first three aspects of health equity talk reflect different understandings of health equity rooted in vulnerability (individual versus structural), determinants of health (material versus social determinants), and appropriate health system responses (targeted versus universal responses). Participants identified that talking about health equity in the health care system, either inside or outside of public health, is a ‘challenging conversation’ because health equity is understood in diverse ways and there is little guidance available to apply a health equity lens.

**Conclusions:**

These findings reflect the importance of creating a shared understanding of health equity within public health systems, and providing guidance and clarity as to the meaning and application of a health equity lens. A health equity lens for public health should capture both the production and distribution of health inequities and link to social justice to inform action.

## 1. Introduction

There has been growing recognition that despite improvements in health care, significant health inequities exist within and between countries. This has been coined ‘the health equity gap’ [1],[2]. Reducing health inequities within and between countries is an ethical, social, and economic imperative and a goal of health systems worldwide [1]–[3]. To close the health equity gap, strong emphasis is being placed on the need to strengthen, reorient and transform health systems towards health equity [2],[4]. In 2011, Canada (and other United Nations member states) endorsed the Rio Declaration on the Social Determinants of Health, which reiterated that a key strategy for achieving social and health equity is the reorientation of the health care sector towards action on the social determinants of health with health equity as a central goal [5]. According to Whitehead and Dahlgren [3], “equity in health implies that ideally everyone could attain their full health potential and that no one should be disadvantaged from achieving this potential because of their social position or other socially determined circumstance.” Differences in age, gender, ethnicity, class, race, social class, religion, socioeconomic status or other socially determined circumstances should not impact resources and opportunities for health [6].

Public health is charged with an important leadership role to address the ‘roots’ or ‘structural determinants’ of health inequities and promote health equity [7]. However, as Baum et al. [4] observe, reorientation is not for the ‘faint hearted’ because health systems are based on ideological values about worth and value and are inherently political [4]. How public health leaders understand health equity is important to how they act to re-orientate health systems towards health equity [8]–[10]. In this paper, we examine senior public health leaders' understandings of health equity and application of a health equity lens during a time of public health renewal in one Canadian province.

Health inequities are unfair, systematic and socially produced injustices that are rooted in social arrangements that disadvantage some groups in the population more than others in terms of health outcomes and opportunities [3],[11],[12]. Health equity and the social determinants of health are therefore linked. The WHO states, “[t]he social determinants of health are the conditions in which people are born, grow, live, work and age. These circumstances are shaped by the distribution of money, power and resources at global, national and local levels. The social determinants of health are mostly responsible for health inequities—the unfair and avoidable differences in health status seen within and between countries [13].”

While health equity has been repeatedly endorsed as central to the mission of public health, health systems are rarely orientated to health equity [10],[14]. Several Canadian reports have recommended strengthening public health infrastructures, supporting effective delivery of public health services, and collaborating beyond public health with the health care system at large and other sectors, to achieve common health goals including health equity [15]–[18].

In British Columbia (BC), Canada, significant policy developments in the past decade have influenced a growing emphasis on health equity during a time of reorganization of public health services within the BC health system. Public health renewal efforts specifically incorporated equity considerations in the development of the “Framework for Core Functions in Public Health” and identified the application of a population lens and an equity lens as integral to meeting the health needs of the overall population [19],[20]. Later, the development of the current Guiding Framework for Public Health continued to endorse and highlight the importance of applying a health equity lens in public health programs and services [21]. Of paramount importance to these efforts is that public health leaders and practitioners have a clear and shared understanding of health equity to effectively implement and work towards reorientation of public health programs and systems.

Among public health leaders, there are different understandings of health equity and the social determinants of health, with varying degrees of attention to root causes and political dimensions of health equity work [8],[12],[22],[23]. For example, among public health leaders in Ontario, Canada, Brassolotto, Raphael and Baldero identified three different understandings of the social determinants of health: functional, structural, and analytic perspectives [24]. A functional approach involves understanding the social determinants of health as identifiers of those people in need of health and social services, as well as those with modifiable medical and behavioural risk factors [23]. An analytical approach acknowledges the multiple ways social determinants impact health and addresses these broader issues within the realm of public health practice [23]. A structural approach includes the functional and analytical perspectives but takes them further to engage with issues of racism, classism, and sexism and how they impact health [23]. These perspectives subsequently inform different actions and approaches to addressing the social determinants of health and thus health equity. For example, activities to promote health equity within a functional understanding might focus on service delivery and lifestyle change; in an analytical understanding, activities may include partnering with community organizations that address issues such as poverty, food security and housing. Finally, within a structural understanding, public health activities may further include public education about the social determinants of health and direct public policy advocacy to economic and political structures as well as power imbalances [23]. Clearly, the latter understanding is aligned with a critical perspective that attempts to address the roots of health inequities.

The *Equity Lens in Public Health* (ELPH) program of research was implemented to study and facilitate learning about the use of an equity lens during a period of complex system change in public health in BC, with an aim to inform systemic responses for reducing health inequities [25]. The ELPH program of research consists of four interrelated studies. The primary goal of one of the four studies is to understand the meaning of health equity, whether it is a priority in the health system, and factors that influence prioritization. Beginning in 2011, the ELPH project has been immersed in a ‘natural experiment’ in which the process of health equity implementation has allowed for an integrated and evolving knowledge translation and exchange (KTE) process [25]. As part of the ELPH research project, we explored how public health decision makers and practitioners in the British Columbia health system understand, prioritize, define and apply a health equity lens in their work during this time of system reorientation.

The ELPH program of research is grounded in critical perspectives on intersectionality, complexity science, and social justice theories [26]–[28]. Intersectionality has emerged as an important theoretical approach to consider the intersections of age, gender, ethnicity, class, ability, sexual identity, and other dimensions of difference in understanding the pathways that influence access to the social determinants of health and the development of health inequities [28]–[30]. The promise of intersectionality lies in theorizing multiple and intersecting identities and how these identities are constituted and situated in the production of inequities through social relations of power enacted through racism, sexism, gender bias, classism and other forms of stigma and discrimination. There are multiple and complex intersections that are multiplicative producing health inequities and impacting resources for health [31]. However, approaches to intersectional analysis often privilege dominant forms of identity (most often class) [27]. Further, although intersectionality may allow one to envision multiple intersecting identities and sources of oppression, it often falls short of theorizing intersections of these oppressions with health and social systems [32].

Walby proposes that intersectionality is compatible with complexity thinking and that complexity theory can be extended to include intersectional perspectives at the systems level [28]. “Complexity theory draws on insights developed in many disciplines and offers the potential of new ways of thinking about social systems and the complexity of their interactions, reworking the understanding of causality” [26]. Walby distinguishes sets of social relations (regimes) and institutional structures (domains). She provides a framework for theorizing about the intersections of multiple social relations (social positioning and systemic processes of stigma and discrimination) and institutional systems including the economy, polity and civil society that impact the development of health inequities and system responses. Walby argues for consideration of both social relations and institutional structures that operate to produce inequities and the intersection of these at multiple levels (individual, group and system). Consistent with a complex adaptive systems (CAS) perspective, Walby [27] rejects the reductionist notion that parts make up a whole system to avoid “reducing to the micro-level of agency or the macro level of structure”. Rather, she argues that each system takes all other systems as its environment. In true complexity fashion these are mutually influential.

Drawing on complexity theory, Walby highlights how institutional arrangements and political agendas intersect with sets of social relations at multiple levels of identity and sources of oppression. Thus, intersectionality theory from a complexity perspective, as described by Walby, allows understanding of sets of social relations that produce inequities. Furthermore, the intersection of these social relations with institutions and political agendas provides guidance in how to address inequities through system responses. Here we conceptualize systems as dynamic, non-linear, evolving, changing, and learning; and the relationships between structure and agency, as co-extensive rather than distinct. We use intersectionality and complexity to analyze health leaders' conceptions of health equity to illuminate some of the intersecting social relations and institutional structures important to the application of a health equity lens.

## 2. Materials and Methods

Our collaborative research team is composed of academic researchers and public health systems leaders from six health regions and the Ministry of Health in BC. Since 2006 we have collaborated on identifying and studying research priorities and were thus poised to study the application of an equity lens in public health. In particular, we have focused on the important but often-overlooked and little understood human component, which is necessary for effective implementation [33].

As part of the collaborative ELPH program of research [25], semi-structured qualitative focus groups and interviews were conducted with senior leaders from six health authorities in BC and the provincial Ministry of Health in 2013/14. These data were collected for a baseline assessment to identify and understand the contextual influences on the uptake of health equity as a priority in the health system and, specifically in relation to the core public health programs of promoting mental health, preventing mental disorders, and preventing the harms of substance use. Ethical approval for this study was provided by the following research ethics review boards: the University of Victoria, the University of British Columbia, the University of Saskatchewan, Interior Health Authority, Island Health Authority, Northern Health Authority, Vancouver Coastal Health Authority, and Fraser Health Authority.

### 2.1. Study Sample

The sample for this study comprises provincial and regional public health staff who were considered to be senior decision makers in their respective organizations and portfolios. These included 55 participants who held roles as senior executives, public health and regional directors, and medical health officers. They participated in 16 individual interviews and 15 focus groups. Written informed consent was obtained prior to conducting the interviews and focus groups.

### 2.2. Analysis

All interviews and focus groups were audio-recorded and transcribed verbatim. All textual data were entered into the computer software program, NVivo™ 2010. Data were analyzed in an inductive and non-linear way [33]. Initially, several reviews of the entire data set were conducted using a constant comparative approach to identify important and interesting features of the data [34],[35]. A coding framework was developed based on these initial reviews of the entire data set to begin to organize the data into meaningful categories. The transcripts were coded using this framework and codes were examined and compared to identify recurring themes in participants' understandings of health equity. We identified trends across all cases, as well as alternative instances that deviated from the dominant themes. The coding framework was further developed and refined throughout the analysis to fully capture emerging themes and alternatives. Specifically, data were analyzed for participants' constructions of the term health equity, with particular attention to the implicit and explicit assumptions inherent in those constructions and the way these constructions reflected Walbys' framework of social relations (regimes), institutional structures (domains) and their intersections.

## 3. Results

All participants in this study felt that health equity was a worthwhile endeavor and considered it important to them as health leaders and professionals. When talking about health equity, most definitions rested on *in*equity as an anchor for definition, rather than equity. Participants reflected their understandings of the sources or production of inequities and/or responses to reducing health inequities. In these results, we reflect on how sets of social relations and institutional domains (particularly political intersectionality) are constructed in the health equity talk of senior health leaders. Within the three themes of population health, determinants of health, and accessibility, we capture how identities, oppressions, and the production of health inequities are constructed and how the political project of promoting health equity is understood within BC's health care system. In a fourth theme of health equity talk, we explore how sets of social relations and institutional domains act as reciprocal environments for each other through examination of the challenges of talking about health equity work within both public health and health systems more broadly.

### 3.1. Population health: Production of health inequities and responses

Many of the participants commented that they do not typically discuss health equity as an explicit term, but that they consider it an implicit concern in population health discussions. Within discussions of population health, participants explicated understandings of vulnerable social groups and gaps between different social groups in the population as a means of identifying who is impacted by health inequities in the population. Health equity work was constructed within discussions of population health as directed at: (1) ‘targeted, vulnerable or at risk populations’; and (2) ‘closing the gap’.

#### 3.1.1. ‘Targeted’, ‘vulnerable’ and ’at risk’ populations

When asked to define health equity, respondents frequently pointed to the importance of health programs or services that were targeted at specific populations, often described as “special”, “at risk” or “vulnerable” as proxies for those experiencing health inequities:

So, frontline Public Health Nurses here rarely use the term health equity. They talk more in terms of at risk populations and have targeted programs for those at risk. (FG 403)

The use of proxies such as ‘vulnerable’ and ‘at risk’ were often used as catch-all terms to encompass a broad range of inequities that were rarely specified in terms of the complex set of social relations operating in the production of health inequities. Although the participant below mentions historical conditions, they quickly turned to unmet health care needs and naming specific populations.

When I hear that term I think of populations who are historically, or due to circumstances, underserved by main stream health services for a variety of reasons and have unmet health care needs because of it. Thinking about Aboriginal populations, street involved populations, people with long term mental illness. (FG 401)

The naming of populations was an attempt to identify social groups that were experiencing heath inequities. Rarely, however, were the intersections of age, race, ethnicity, gender/sex and class identified or how these identities are constituted within sets of social relations and the role of racism, sexism, classism and other forms of discrimination identified as the sources of health inequities. Although at least one participant pointed out that discussions around the intersections of inequities more often occurred within women's health discussions:

But what I would say is within women's health… those discussions have been going on forever…it's slightly more…there's a number of layers. And so gender, diversity, education…you kind of layer in everything into the discussion in the women's health world, so I find it really a different kind of discussion. (FG 702)

This participant highlighted that women's health discussions often foreground sex and gender and the multiple intersecting identities and determinants of health that impact health equity.

In the context of discussions of vulnerable or at risk populations, participants discussed targeted programs as a health equity response for ‘vulnerable’ or ‘at risk’ populations. However, there was often confusion between a truly targeted program (which was available to only a specific population), versus enhanced services within already existing, universally-accessible programs. In the following quote, the participant first described providing a targeted program that is focused on a specific population, but then described tilting or enhancing services for a specific group within a larger program, which would be more in line with proportionate universality or enhanced services.

We work on programs that are very much oriented towards health equity or with the intention of reducing inequities, but I don't think we use that type of language. The phrase ‘health equity’ may not even come up, even though it's understood that the program is focused on a portion of the population that may be at particular high risk of a disease or of a particular condition…we would say “What is the population? What are their needs?” and then we would measure whether we were meeting their needs or not. If we have to tilt programs to meet the needs of one group in a different way than others, that would be, you know, providing equity. (FG 601)

Regardless of the level of understanding surrounding targeting vulnerable groups versus proportionate universalism, implicit in many of these understandings was a focus on identifying vulnerable individuals, groups or populations rather than the conditions that produce vulnerability. Notably, one participant expressed the tension between vulnerable populations and structural conditions that create vulnerability.

But I think that, now, if equity is going to be a…if there's traction there, then from a society value piece than we need to be…that dialogue needs to be broad in terms of ‘what's our responsibility to the vulnerable segments of our population?’ and ‘why are some segments of the population vulnerable?’ is it their doing, or is it the way that things are structured? And it's those conversations that I think are still very challenging. (FG 405)

This participant provided a more nuanced understanding of vulnerability and the importance of structural vulnerability both in the development of health inequities and actions to promote health equity. However, populations were rarely defined beyond an assigned label of being “at risk” or “vulnerable”, implying that these statuses were understood as inevitable or inherent for certain groups of people, and therefore ignoring the roles of institutions and social relations of power in creating health inequities in the first place.

#### 3.1.2. Closing the gap

Discussions around the fairness and causes of differences in population health often turned to discussing health equity in relation to closing the gap between health statuses of different groups and populations. Many participants described their understanding of health equity by pointing to the fact that health status varies according to a social gradient, and that there are considerable differences between the health of some groups compared to others. For these participants, health equity was explicitly concerned with closing the gaps between groups:

Making sure that the services we deliver reduce gaps, avoidable gaps in health status, are fair and equitable across the health region, but health equity means we are reducing or eliminating differences in health status or health outcomes that are either preventable or avoidable or that are un, that we think are unjust. Not all differences in health are avoidable or unjust. You know, there are genetic differences and what have you, but we are trying to address those differences that we think shouldn't be there. (FG 302)

This participant focuses on ensuring that services reduce or eliminate differences in health status that are preventable or avoidable. The participant below highlighted the differences in health status that arise depending on how data are disaggregated and the challenge in making value judgements about what inequities are unfair and unjust as well as amenable to an intervention.

[W]hen we look at averages, the average male or female life expectancy is x but then if you disaggregate that by regional basis or your economic status or by educational status, it's clearly different. And some of that health status might be inherent or biological or just bad luck and there's not much you can do about it. But some of it is amenable to presumably some other kind of intervention. But it's really a value judgment, whether something is fair or unfair I think. So I think the challenge is addressing those value judgments at a policy level or at a systemic level and that's what- and then, when you're talking about targeting proportional universality you're talking about addressing inequities or unfairness and by raising awareness of them and then deliberately targeting to address a gap. A concept of fairness [is] embodied [in] inequity, which you don't find in inequality. (FG 702)

What is interesting and important about discussions of closing the gap is that while such discussions highlighted important differences in health status they also focused on what intersections should be explored and the political nature of making the judgements necessary to inform appropriate interventions. What is not clear is whether such decisions were based on an understanding of social relations of power and the role of racism, sexism, classes and other ‘isms’ in the production of health inequities.

### 3.2. Determinants of health

Although there were challenges and limitations to understanding the complexities of multiple sets of social relations and institutional responses through a population health lens, participants recognized that the conditions to support health are not distributed equally and that the social determinants of health play an important role. Participants focused on material versus social determinants of health and distinctions between individual and structural foci of action on the social determinants of health.

#### 3.2.1. Material versus Social determinants of health

The ‘determinants of health’ was used by many participants as a proxy term for health equity. Determinants were broadly defined by participants to include ‘income’, ‘education’, ‘rurality’, ‘housing’, ‘policy’, ‘justice’, ‘literacy’, ‘food security’, and ‘housing security’. As one participant explained, these determinants were often considered the root causes of health inequity:

I think that when we talked about equity, or inequities--people- there're a lot of belief and buy in into the fact that you can't deal with this downstream. You need to deal with the root causes and you need to deal with the social determinants and you need to deal with all of that upstream stuff. (FG 601)

In describing and discussing determinants of health, material determinants of health such as housing, income, education, and food were most often identified:

But I think that if we look at the links between health equity and the whole social determinants side and […] if we understand and accept the relevance of social determinants, then we say, well of course it's a no-brainer, that safe and secure housing must be a consideration somewhere in the dialogue, [same] as income and resources […] [But,] I think that very often those…appear in the dialogue too big to tackle. (FG 405)

As this participant pointed out, even considering the material determinants of health is too big and challenging to tackle within the health care system, let alone trying to understand how the availability of determinants like housing, income and other resources are different for many based on their social locations (e.g., race, gender, age, ethnicity, class, sexual orientation) within the social system. Yet, bringing an understanding of social relations of power such as racism, sexism, gender bias, ableism or classism and the intersections of these in the production of inequities could illuminate the importance of respect, power, and decision making as important to addressing health inequities within the health care system. Thus, participants rarely discussed strategies related to addressing systemic processes of stigma and discrimination or engagement and involvement of those with less power and resources in their work to reduce inequities. Even though meaningful involvement is central to health promotion practice [36],[37].

#### 3.2.2. Action on individual versus structural determinants of health

Participants understood the determinants of health as the preconditions for health equity as well as the sources of health inequities, but conceptualized this distribution at either the individual or the structural level. As a result, the main concern for supporting health equity was a focus on health care resources (including promoting healthy choices and lifestyles) and other services, and on those people experiencing the inequity to increase their individual access to these resources.

And then looking at that reality and then looking at a service array that's really focused on treating mental illness, but not necessarily treating the whole person or preventing chronic disease in a population that we know is at particularly elevated risks for chronic disease. So we're starting to develop program initiatives around that, that is much more of a holistic and forward looking approaches rather than just treating the most obvious problem being the mental illness but missing what is going to rob that person of a lot of years of life if it's not addressed concurrently in a preventative way. (FG 401)

In this instance, the preventative way meant ensuring that individuals have access to determinants of health and healthy choices thus focusing on the individual rather than addressing structural change. Conversely, some participants identified the need to understand how populations are positioned in relation to the determinants of health and their ability to take up and access various determinants.

So, some populations, as a result of the social determinants of health, are very well positioned and advantaged to experience optimal health outcomes as a result whereas some are actually in a position, because of a number of factors, that may be social or environmental, or perhaps genetic in context, are in a situation where they actually are not very well positioned to experience optimal health outcomes. And so as a result, what we need to do, and I'm thinking about policy interventions or [problem based] interventions, or system based interventions, we need to be considering where the opportunities are to account for those factors with those elements that are contributing to setting a population up to experience less than optimal outcomes. So we're going to compensate some way through our efforts in order to ensure that they're in a place where they can experience health outcomes that are equitable to others in the population who may be in a position that they're actually well positioned or advantaged to experience. (FG 705)

This participant identified that system-based interventions are needed to mitigate sets of social relations that produce health inequities and important for everyone to be positioned to access resources for health. As the participant below points out, we need to go further and connect this to understanding inequities as systemic injustices thus related to social justice.

If we really understood health equity and health inequity, and that it's actually fundamentally rooted in differences that are avoidable, and due to injustice--like there's …a very significant social justice component. (FG 104)

Inequities are often produced outside of the health system, which highlights the importance of recognizing external policy and political structures. Most participants agreed that action on the determinants of health would lead to improved health equity but neglected, as discussed earlier, to acknowledge the impacts or importance of issues related to sets of power relations that produce racism and discrimination within the health care system itself as sources of health inequities.

### 3.3. Accessibility

In participants' views, ensuring accessibility of health services and resources for health was a key health system response for addressing health inequities. Participants understood accessibility in three distinct ways, namely as: equal availability of health services, equitable access to health services, or equal outcomes through unequal health services.

#### 3.3.1. Equal availability of health services

The equality discourse was a prominent undercurrent in how participants discussed health equity, with equality and equity frequently confounded to mean the same thing. Within discussions of equality, participants discussed that everyone should be treated the same and have equal availability of health care services. Universal health care and programs were cited as examples of strategies that support health equity, and participants stated that this ensured equal access to health services for everyone. Importantly, equal availability to health care services specifically was stressed, not resources generally, because the sources of health inequity were implicitly constructed as a lack of health care usage and access. Within this theme, access to services was emphasized to mean solely that the services were available and that people hypothetically had the opportunity to access them, rather than actual access to services.

I would say it's not even equity of access. I think it is more equal opportunity to access. So in other words, if you show up at the hospital and you get in the line at the queue, you have equal opportunity to be seen as someone else.” (FG 104)

Although participants discussed universal health care and programs as examples of addressing health equity, they also recognized that these programs and services were not distributed equally throughout BC despite universal publically funded health care in Canada. So, health equity was understood as having equal services in all regions of BC so that similar types and quality of health services could be accessed regardless of geographical location. These discussions further highlighted values of sameness or equality of service availability, regardless of differences in geography or other structural differences, while remaining purely topical in terms of a deeper understanding of equitable access. Thus, an equality of services approach focuses on a health system response but misses the importance of intersections between having health services available and the ability of people to access these services given their social location and multiple intersecting oppressions that may prevent access.

#### 3.3.2. Equitable access to health services

The concept of equitable access rested on the idea that simply providing a health care service was not sufficient to support health equity. Rather, participants focused on removing barriers to provide either equitable access to health services or to support equal outcomes after accessing a health care service. Again, and importantly, this theme was focused squarely on health care services, rather than broader resources or determinants of health, with the implication that equitable access to health care services is considered the solution to supporting health equity.

Many participants related the term health equity to ensuring access to health services, regardless of who people are or where they live geographically. Participants pointed out that simply providing a service was not sufficient to support health equity. These participants discussed access in terms of removing barriers to health services to support health equity. Examples of ways to increase equity in access to health services included having more flexible hours, providing transportation and/or childcare, doing outreach work and home visits, removing the need for referrals to access services, and providing non-judgemental care. These types of understandings often included supporting health equity through providing care that reflected the unique needs of the client, rather than providing equal services across the board:

I was just thinking of equity of service,…. and to me, it might be different than somebody from an Aboriginal community, and their richness in regards to their traditional medicine and accessing help in regards to, residential schools and stuff. My definition of them having access to services might be different than what theirs is. And, their service- equity of services- that it's provided there amongst people that are familiar with their traditions and their values, and their culture. (FG 502)

This quote highlights the importance of communities being involved in defining what is meant by equitable in terms of the type, focus and nature of the services.

#### 3.3.3. Equal Outcomes through Unequal Services

Some participants acknowledged that due to multiple intersecting oppressions, people are often positioned differently in their ability to access resources. These participants noted that health equity is not just about access to health services, but whether there are equal health outcomes once health services have been accessed:

I think in our health care system, and health system, we're used to working under the guidelines of the Canada Health Act and very comfortable with treating everybody equally, but health equity to a certain extent means not treating everybody equally. So I think sometimes it's that difference, that you know, we want equitable outcomes, not necessarily, [equal] process or treatment in how we interact with our populations and patients. (FG 202)

These participants were adamant that health equity was not solely about accessing services, but whether services were accessible, and whether or not such there were equitable health outcomes across the population and geographies of the province:

That would be every government's line, everyone has equal, you know, everyone is treated equally. Well actually, that's…that's- yes, everyone is equal under the law, doesn't mean that the outcome is equal. Though, in fact, you have to provide different amounts of input at the front end to end up with equal outcomes. And that's what really we should be talking about is equal outcomes in health care. Are people able to have the…the…equal outcomes and if you can achieve that, that is a health equity around a particular topic. So, trying to reframe people's thinking away from the front end to actually the back end, and the outcome, is really important. (INTV 407)

What is well illustrated in these quotes above is an understanding that there is the expectation that everyone should be treated equally, but that equal treatment is not adequate for achieving equal outcomes. Achieving equal outcomes in health therefore requires that some receive more resources and services than others. This moves towards an understanding of proportionate universality [40] as a response that seeks to reduce health inequities across the social gradient. This highlights important and complex relationships between understanding how individuals, groups and populations are situated in relation to multiple intersecting identities and the structural or institutional arrangements that govern the determinants of health including access to healthcare. As some participants above observed, engaging communities is an important strategy for addressing health inequities.

### 3.4. Health equity talk: Challenging conversations

The overall sentiment, as outlined by some of the participants above, was that talking about health equity was a challenging conversation due to the complexity of understanding and communicating about health equity. In this section, we share participants' perspectives on the complex nature of health equity and the difficulties of linking health equity with action as well the importance of grounding health equity in social justice if there is to be effective action on health inequities.

#### 3.4.1. We don't have a shared understanding

Participants talked about difficulties with defining the term health equity and felt that it had become an umbrella term that was gaining momentum without clarity as to its definition or application. The participant below pointed to the complexity of defining health equity:

The reason it's not a good discussion is all to do with definition of the term. I think we have to be, as people who are concerned about health equity, in its multi-dimensional, complex kind of, like the whole ball of wax. (INTV 704)

This quote illustrates the complexity of defining health equity. We concur that it is often difficult, as demonstrated by the previous themes, to capture understandings of the complex intersections of social relations and institutional domains and the challenge of illuminating and analyzing complex intersections and systems responses. In addition, as demonstrated above, participants stated that health equity was often discussed through the use of proxy terms or ideas such as vulnerable populations and social determinants of health, which were meant to imply the concept of health equity but without the necessary attention to social relations and institutional domains that contribute to the production of inequities.

In addition to not always having a shared understanding of health equity within public health, there was the issue of talking about health equity outside of public health in the broader health care system. When participants from public health discussed health equity with those from other parts of the health care system, most notably the acute care system, problematic and competing discourses became evident. Many of our participants found that “we're speaking different languages” because of a lack of or diverse understandings of health equity held by people in different parts of the health care system. Most participants are grappling with how to understand the complex intersections in the production of health inequities and therefore with how the system should respond. Thus, they found it difficult to communicate about health equity and influence value shifts in the health care system.

#### 3.4.2. Connecting health equity to social justice

Many of the public health participants felt that health equity was just another fad term to describe work that public health had always been doing but was named differently as the popular term of the day. As the participant points out below, health equity is really just concerned with justice but has been packaged into a new term:

I would say I'm not sure what health equity is, as any kind of a term… so, equity is really a financial term. Right? And then we've translated it into health as saying, well, it's health equity. And then people like to put the ‘in’ in front of it, and now it's health ‘in’equity. And we've taken, and all this is in the guise in my mind, of it's a backdoor way of trying to talk about justice. Right? And, and so, what are we really talking about? (INTV 701)

When participants discussed health equity in relation to social justice, they were more able to engage critically with concepts of health and seemed to feel more empowered, not only about their understanding of health equity, but also about the role they felt they, and the health system, were playing or could play in reducing health inequities:

The language that I like is this idea of looking at the health system trying to [address]… things that are avoidable, that to me, that's a big piece in health equity. Like, looking at the health system, and issues that—this sort of inequity or inequality that's occurring due to sort of avoidable issues—things that we can do within the health system that really…inequities that…are within our control. (INTV 607)

By understanding health equity through the frame of justice or social justice, participants appeared more comfortable to speak about the term, hypothesize on its application and identify having a role to play in relation to it. This was likely due to the fact that for many working in public health or the health sector, social justice is already considered a cornerstone of their values and work:

Well, we had a bit of a discussion earlier about whether one of our operating principles was social justice. And I think for me, equity is about fairness and about social justice and that everyone is entitled to good health, and, therefore all the things that will enable good health. (FG 705)

Participants wanted more information on how they could actually apply health equity concepts in a practical way to their day to day work to enact social justice: “Yeah, I find that a lot of people I talk to intuitively understand health inequity but can't think of how to apply it”. Further, some participants thought that the lack of practical tools for applying it discouraged people from actively engaging with health equity:

So you don't really talk in terms of “We're going to work to reduce health inequities”. That may be actually symbolic of our understanding, is that you need practical tools. You need practical things. You don't…go around flaunting health equity because I don't think most people would know what the heck it was. (FG 501)

As well, for many participants, the discussions around health equity were often too far removed from the people actually experiencing inequity:

Health equity isn't going to be anything more than this decade's ‘phrase’ if we don't actually make sure that the people we're talking about are actually in the conversation. (FG 501)

This participant made clear the importance of community engagement and including communities that are often missing from conversations about promoting health equity. They see this as an important strategy for promoting social justice [2],[38],[39].

## 4. Discussion

Across BC health authorities, participants identified that there was not a shared or common understanding of health equity, with participants focusing on different aspects of health equity including population health, determinants of health and accessibility. Participants reported that the application of a health equity lens is a challenge because health equity is understood in diverse ways, is often not linked to social justice with little practical guidance about how an equity lens can be applied. Their experiences highlight that implementation of a health equity lens is complex and that it is difficult within health systems to capture fully the understandings of complex social relations that produce inequities as well as how health systems can and should response to health inequities.

The construction of health equity as a population lens that focuses on vulnerable and marginalized populations or gaps in health status is limited in that vulnerability is often located within an individual or group rather than in the conditions that created injustices in the first place [38]. The use of ‘vulnerable’ and ‘at risk’ decontextualizes the social determinants of health from broader public policy approaches and structural inequalities [8]. Naming groups or populations as vulnerable and/or marginalized without acknowledgement of the structural conditions that contributed to inequitable health outcomes in the first place has the potential to further marginalize and stigmatize populations. There is a high level of complexity in understanding, measuring and planning health services that are sensitive to social relations of power and how responses are constructed within current health and social systems. As discussed by participants, proportionate universalism has a broader reach in relation to those experiencing structural vulnerability as opposed to targeting only populations deemed to be vulnerable [40]. We highlight the importance of shifting understandings of vulnerability away from individual (naming of groups and populations) to structural vulnerability in which social conditions and institutional arrangements are implicated [41].

Many participants indicated that determinants of health are important in driving health inequities and articulated determinants of health as important to understanding and promoting health equity. However, in keeping with a prominent critique of population health research in general, an over-reliance on applying unitary determinants across broad segments of a population is a reductionist understanding of complex social relations that have multiple intersections with institutional domains [42]. As Bauer points out, this conflation of related constructs into the lowest common denominator to explain health inequalities of population groups “may serve to reinforce existing notions of intractability of injustice, while failing to identify intervenable factors that might be candidates for potential solutions” [42]. Indeed, material determinants of health such as income, education, and housing were mentioned often, highlighting class or socio-economic status. However, participants rarely mentioned important social determinants such as racism, sexism, classism, and abelism [43]–[45] or important processes such as respect, power and decision making [38],[46]. Participants did highlight the importance of involving community but without recognition of inequitable power relationships that shape such involvement of communities impacted by inequities. This points to the importance of greater recognition and awareness within public health of the relationship between social positioning and systemic processes of racism, sexism, classism, abelism and other forms of discrimination as key social determinants of health as well as the importance of respect, power and decision making as key strategies for promoting health equity.

Discussions related to the intersections of these determinants, which could accommodate the fluid and interactive nature of the multi-level complex processes and systems that shape health inequities, were virtually absent [47]. These omissions are particularly salient in the BC context where sex and gender has been shown to explain differences in health care access over and above socioeconomic status [48] and Indigenous peoples in BC and Canada continue to experience serious and ongoing health inequities associated with colonization, racialization and experiences of racism in the health care system [28]. While Indigenous peoples were often named as a population experiencing inequities, participants did not actually name racism or colonialism as root causes of this inequity. Racism only becomes a determinant if we acknowledge the conditions, rather than the population, and this depth of discussion was often missing from discussions around defined populations in relation to health equity. Importantly, while participants recognized the need for health system responses, the health system itself was not readily included as a determinant of health nor was the role of public health, in either contributing to or reducing stigma and discrimination in the health care system, explicitly acknowledged as part of a strategy to promote health equity.

As Brassolotto, Raphael and Baldero identify, the lack of engaging with health equity as a political issue is a limitation in addressing determinants of health [8]. Public health practitioners face challenges in promoting health equity in an era of institutional constraints on their ability to advocate for policies outside of health, and the pressure on and in health systems to be apolitical. This may, in part, explain the lack of focus on intersections of social relations of power and institutional domains that are politically rooted. Positively, these findings did not highlight health equity as focusing on individual lifestyle change, or as some have described, ‘lifestyle drift’. Individualism, as a discourse rooted in neo-liberalism, often dominates health, and while participants did not focus on lifestyle modifications, their focus on individual access to the social determinants of health still reflected values of individualism in health.

“Service utilization” as a measure of access is problematic. Access is “multidimensional” and the importance of unequal services for equal health outcomes was captured by some participants and highlighted the importance of proportionate universalism rather than merely targeting groups [48]. Inequity is reflected in unmet needs, but requires multi-dimensional understandings which makes grasping and talking about health equity as access to service limited to conditions that shape access to health care, not health [48]. When health is equated with health care, understandings of this complexity are limited and public health can readily be subsumed by acute care. This obscures the need for health system reorientation to value and address health equity and the need to shift funding from acute care to public health to operationalize health equity actions [49].

There is a growing call to action for health promotion and social determinants of health approaches to move towards social justice as an explicitly political endeavour and for public health action to challenge existing and historical distributions of power and resources to structurally and effectively reduce health inequities [50]. This shift, which will require multi-level and multi-sectoral involvement, is paramount to overcoming limits to health equity understandings and application in public health settings. In particular, as noted by participants, health system responses are often distant from meaningful participation of communities, which are inherently political and central to endeavors of social justice and to discussions of power and resources [2],[51].

Illuminating the link between social justice and health equity is an important insight that aligns with complexity theory and the emergence of new and evolving understanding [26]. Walby suggests that it is often difficult for sets of social relations and institutional structures to be fully exploited but analyzing these separately and highlighting the complex intersections between them is important for understanding social inequalities [27],[28]. Given that social inequalities are the root causes of health inequities [52], we propose that examining the production of health inequities through an intersectionality lens is useful for illuminating sets of social relations that produce inequities, but that a health equity lens also requires an understanding of social justice that specifically focuses on responses of institutional systems in mitigating inequities and promoting health equity. We conclude that a health equity lens informed by intersectionality, critical social justice theories and complexity holds potential to illuminate unfair, systemic and social production of health inequities and inform action to reduce health inequities and promote health equity [32].

### Strengths and limitations

This research is part of larger body of research on health equity that includes data from health leaders identified as decision makers during a time of complex public health system change in BC health authorities. These findings highlight baseline understandings of health equity for health leaders more than eight years after it was recommended that a health equity lens be applied to public health programs in BC. Thus, these findings are representative of the time period during the initial implementation of a health equity lens, but understandings may have subsequently shifted. As well, the findings are not restricted to public health leaders but also reflect the views of a broader set of health system leaders who may not have a background in public health. Semi-structured interviews and focus groups were conducted by experienced interviewers, which allowed ideas to be explored in depth. However, in focus groups, some participants may not have felt comfortable articulating their thoughts or feelings if the group was dominated by someone with whom they worked or perceived had more power.

## 5. Conclusions

Definitions of health equity varied, reflecting different understandings of vulnerability (structural versus individual), determinants of health (material versus social determinants), and appropriate health system responses (targeted versus proportionate universalism, individual versus structural interventions). Applying the theoretical perspectives of intersectionality, complexity and critical social justice allowed insight into potential constructions and limitations of health equity understandings and important shifts needed for health systems reorientation towards health equity. Findings reflect potential strengths and challenges for reducing health inequities in health systems and that a health equity lens that encompasses the production and distribution of health inequities through complex and intersecting sets of social relations and instituitonal structures to inform health systems responses rooted in social justice is needed for health systems reorientation towards health equity.

## References

[1] Crombie I, Irvine L, Elliott L (2005). Closing the health inequalities gap: An international perspective.

[2] Commission on the Social Determinants of Health (2008). Closing the gap in a generation: achieving health equity through action on the social determinants of health.

[3] Whitehead M, Dahlgren G (2006). Levelling up (part 1): A discussion paper on concepts and principles for tackling social inequities in health.

[4] Baum F, Begin M, Houweling T (2009). Changes not for the fainthearted: reorienting health care systems toward health equity through action on the social determinants of health. Am J Public Health.

[5] Stankiewicz A, Herel M, DesMeules M (2015). Report summary--Rio political declaration on social determinants of health: a snapshot of Canadian actions 2015. Health Promot Chronic Dis Prev Can: Res Policy Pract.

[6] National Collaborating Centre for Determinants of Health (2013). Let's talk: health equity.

[7] Sadana R, Blas E (2013). What can public health programs do to improve health equity?. Public Health Rep.

[8] Brassolotto J, Raphael D, Baldeo N (2014). Epistemological barriers to addressing the social determinants of health among public health professionals in Ontario, Canada: a qualitative inquiry. Crit Public Health.

[9] Varcoe C, Pauly BM, Laliberte S, Hankivsky O (2012). Intersectionality, social justice and influencing policy. Health inequities in Canada: Intersectional frameworks and practices.

[10] Raphael D (2003). Barriers to addressing the societal determinants of health: Public health units and poverty in Ontario, Canada. Health Promot Int.

[11] Graham H (2004). Social determinants and their unequal distribution: Clarifying policy understandings. Milbank Q.

[12] Braveman P (2014). What are health disparities and health equity? We need to be clear. Public Health Rep.

[13] World Health Organization (2017). What are the social determinants of health?.

[14] Bryant T, Raphael D, Schrecker T (2011). Canada: A land of missed opportunity for addressing the social determinants of health. Health Policy.

[15] Canadian Institutes of Health Research (2003). The future of public health in Canada: Developing a public health system for the 21st century. Ottawa: Author.

[16] Di Ruggiero E, Frank J, Moloughney B (2004). Strengthen Canada's public health system now. Can J Public Health.

[17] Select Standing Committee on Health (2004). The path to health and wellness: Making British Columbians healthier by 2010.

[18] Bill C (2002). The standing senate committee on social affairs, science and technology. The health of Canadians – the federal role.

[19] Ministry of Health Services Population Health and Wellness (2005). Public health renewal in British Columbia: an overview of core functions in public health.

[20] Ministry of Health Services Population Health and Wellness (2005). A framework for core functions in public health.

[21] British Columbia Ministry of Health (2013). Promote, protect, prevent: Our health begins here. B.C's Guiding Framework for Public Health. Population Health and Wellness BCMoHS.

[22] Braveman P (2006). Health disparities and health equity: concepts and measurement. Annu Rev Public Health.

[23] Smith MJ (2015). Health equity in public health: clarifying our commitment. Public Health Ethics.

[24] Brassolotto J, Raphael D, Baldeo N (2013). Epistemological barriers to addressing the social determinants of health among public health professionals in Ontario, Canada: A qualitative inquiry. Crit Public Health.

[25] Pauly BM, MacDonald M, Hancock T (2013). Reducing health inequities: The contribution of core public health services in BC. BMC Public Health.

[26] Walby S (2007). Complexity theory, systems theory, and multiple intersecting social inequalities. Philos Soc Sci.

[27] Walby S, Armstrong J, Strid S (2012). Intersectionality: Multiple inequalities in social theory. Socioogy.

[28] Walby S (2009). Theorizing Multiple Social Systems. Globalization and Inequalities: Complexity and Contested Modernities.

[29] Hankivsky O, Cormier R (2009). Intersectionality: Moving women's health research and policy forward.

[30] Hankivsky O, Reid C, Cormier R (2010). Exploring the promises of intersectionality for advancing women's health research. Int J Inequity Health.

[31] McCall L (2005). The complexity of intersectionality. J Women Cultur Soc.

[32] McGibbon E, MacPherson C (2011). Applying intersectionality and complexity theory to address the social determinants of women's health. Women's Health Urban Life.

[33] Ostlin P, Schrecker T, Sadana R (2010). Priorities for research on equity and health: Implications for global and national priority setting and the role of WHO to take the health equity research agenda forward. Final report.

[34] Boeije H (2002). A purposeful approach to the constant comparative method in the analysis of qualitative interviews. Qual Quant.

[35] Fram SM (2013). The constant comparative analysis method outside of grounded theory. Qual Rep.

[36] Marent B, Forest R, Nowalk P (2012). Theorizing participation in health promotion: A literature review. Soc Theory Health.

[37] MacDonald M, Mullett J, Potvin L, McQueen DV (2008). Dilemmas in health promotion evaluation: Participation and empowerment. Health promotion evaluation practices in the Americas.

[38] Young IM (1990). Justice and the politics of difference.

[39] Fraser N, Lovel T (2007). Re-framing justice in a globalizing world. (Mis)recognition, social inequality and social justice: Nancy Fraser and Pierre Bourdieu.

[40] Carey G, Crammond B, De Leeuw E (2015). Towards health equity: a framework for the application of proportionate universalism. Int J Equity Health.

[41] Quesada J, Hart L, Bourgois P (2011). Structural vulnerabilty and health: Latino Migrant Laboreres in the United States. Med Anthropol Q.

[42] Bauer GR (2014). Incorporating intersectionality theory into population health research methodology: challenges and the potential to advance health equity. Soc Sci Med.

[43] Priest N, Paradies Y, Trenerry B (2013). A systematic review of studies examining the relationship between reported racism and health and wellbeing for children and young people. Soc Sci Med.

[44] Paradies Y, Jehonathan B, Denson N (2015). Racism as a determinant of health: A systematic review and meta-analysis. PLOS One.

[45] Benoit C, Shumka L (2009). Gendering the population health perspective: Fundamental determinants of women's health.

[46] Powers M, Faden R (2006). Social justice: the moral foundations of public health and health policy.

[47] Hankivsky O (2012). Women's health, men's health, and gender and health: implications of intersectionality. Soc Sci Med.

[48] Socías ME, Koehoorn M, Shoveller J (2016). Gender inequalities in access to health care among adults living in British Columbia, Canada. Women's Health Issues.

[49] Marmot M, Allen JJ (2014). Social determinants of health equity. Slov J Public Health.

[50] Jackson SF, Birn AE, Fawcett SB (2013). Synergy for health equity: integrating health promotion and social determinants of health approaches in and beyond the Americas. Rev Panam Salud Publica.

[51] Solar O, Irwin A (2010). A conceptual framework for action on the social determinants of health. Social Determinants of Health Discussion Paper 2 (Policy and Practice).

[52] Shareck M, Frohlich KL, Poland B (2013). Reducing inequities in health through settings related interventions - a conceptual framework. Glob Health Promot.

